# Inactivation of SARS-CoV-2 on salt-coated surfaces: an in vitro study

**DOI:** 10.1007/s00203-023-03614-9

**Published:** 2023-06-30

**Authors:** Monika Gsell, Xavier Bulliard, Sandra Schorderet Weber, Yang Xiang, Samuel Constant, Sandro Steiner, Silvia Biselli, Raphael Pugin, Michele Palmieri, Andreas Hogg, Manuel C. Peitsch, Julia Hoeng, Adrian Stan

**Affiliations:** 1 Institute for Infectious Diseases, University of Berne, 3010 Bern, Switzerland; 2 Swiss Institute for Translational and Entrepreneurial Medicine, 3010 Berne, Switzerland; 3 Centre Suisse d’Electronique et de Microtechnique SA (CSEM), Rue Jaquet-Droz 1, 2002 Neuchâtel, Switzerland; 4 PMI R&D, Philip Morris Products S.A., Quai Jeanreaud 5, 2000 Neuchâtel, Switzerland; 5 Epithelix Sàrl, Chemmin des Aulx 18, Plan-Les-Ouates, 1228 Geneva, Switzerland; 6 Coat-X SA, Eplatures-Grise 17, 2900 La Chaux-de-Fonds, Switzerland

**Keywords:** SARS-CoV-2, COVID, Antiviral coating, Face masks, 3D lung cell culture

## Abstract

In the COVID-19 pandemic, caused by the severe acute respiratory syndrome coronavirus 2 (SARS-CoV-2), face masks have become a very important safety measure against the main route of transmission of the virus: droplets and aerosols. Concerns that masks contaminated with SARS-CoV-2 infectious particles could be a risk for self-contamination have emerged early in the pandemic as well as solutions to mitigate this risk. The coating of masks with sodium chloride, an antiviral and non-hazardous to health chemical, could be an option for reusable masks. To assess the antiviral properties of salt coatings deposited onto common fabrics by spraying and dipping, the present study established an in vitro bioassay using three-dimensional airway epithelial cell cultures and SARS-CoV-2 virus. Virus particles were given directly on salt-coated material, collected, and added to the cell cultures. Infectious virus particles were measured by plaque forming unit assay and in parallel viral genome copies were quantified over time. Relative to noncoated material, the sodium chloride coating significantly reduced virus replication, confirming the effectiveness of the method to prevent fomite contamination with SARS-CoV-2. In addition, the lung epithelia bioassay proved to be suitable for future evaluation of novel antiviral coatings.

## Introduction

With the coronavirus disease of 2019 (COVID-19) pandemic, governments have developed strategies and guidelines to control the transmission of severe acute respiratory syndrome coronavirus 2 (SARS-CoV-2) in the general population. These guidelines have recommended or mandated the use of face masks in various public places (Center for Disease Control and Prevention (CDC) 2020; European Center for Disease Prevention and Control (ECDC) 2020; Swiss National COVID-19 Science Task Force (NCS-TF) 2020; World Health Organisation (WHO) 2020). Because of the initial shortage of surgical and community disposable face masks in the early days of the pandemic (Wu et al. 2020) but also supported by concerns regarding the sustainability of public measures, the use of reusable homemade or commercially produced face masks has been accepted as a valid alternative solution (European Center for Disease Prevention and Control (ECDC) 2020). As the pandemic progresses, disposable and reusable masks have been used to impede the transmission of upper respiratory diseases. However, concerns regarding the potential risk of self-contamination (World Health Organisation (WHO) 2020) have prompted a debate on whether the general population is more exposed to transmission because of the improper handling of face coverings, such as reusable or disposable (surgical) face masks.

Face mask manufacturers or consumers have proposed a solution that involves coating the surface of face masks with antiviral layers even before the COVID-19 pandemic. Although this proposal is not new, coating the surface of masks with antiviral agents may mitigate the risk of fomite transmission (Chughtai et al. 2019). Various solutions, including antimicrobial polymers, biomolecules, metals, and metal oxides, have been considered for the antiviral coating of face masks (Borkow et al. 2010; Balagna et al. 2020; Pemmada et al. 2020; Pullangott et al. 2021; Takayama et al. 2021; Tunon-Molina et al. 2021). Before the COVID-19 pandemic, Quan et al. (2017) demonstrated a simple do-it-yourself method of coating surgical face masks made of nonwoven polypropylene with NaCl to avoid self-contamination. They found that within 5 min, the H1N1 influenza virus is inactivated when the coating layer wetted with the virus laden aerosols locally dissolves, subsequently evaporates, and recrystallizes. They concluded that these physicochemical processes damage the viral envelope, leading to viral inactivation (Quan et al. 2017). They further reported that NaCl can functionalize inert membranes, causing the efficient capture and inactivation of airborne pathogens (Rubino et al. 2020). The assumption that inactivation through envelope alteration observed with H1N1 (negative-sense single-stranded RNA) could be extended to any enveloped virus even not closely related to influenza viruses has been evaluated in another model using spunlace fabric coated with cranberry extract that affected SARS-CoV-2 (positive single-stranded RNA) and bacteriophage phi6 (double-stranded RNA) in a similar way (Takayama et al. 2021).

Very recently, Schorderet Weber et al. (2022) demonstrated that salt coating applied to common textiles suitable for manufacturing of reusable face masks was able to significantly reduce Influenza H3N2 virus replication in an in vitro lung cell culture system. The aim of this work was to verify that reusable face mask made of nonwoven textiles coated with salt were also able to inactivate SARS-CoV-2. Consequently, working with easily accessible coating methods (spraying and dipping), we tested the coated material for its antiviral properties in the human 3D lung epithelial cell culture bioassay adapted to SARS-CoV-2 infection.

## Materials and methods

### Test material

The test material was a universal cloth (Jemako^®^, Rhede, Germany) made of nonwoven microfibers (80% polyester/20% nylon). It was coated with salt at various concentrations and dried overnight at room temperature (20 °C). It was stored under nitrogen atmosphere in a clean sealed plastic bag until use. Before being tested, the fabric was cut into 1 cm^2^ pieces (three pieces per treatment group) and placed in a sterile 24-well plate. Both sides of the fabric were exposed to ultraviolet C radiation by using a TUV 30W/G30 T8 lamp (Philips Lighting, Signify, Eindhoven, The Netherlands) for 30 min.

### Coating solution

A salt solution containing 29.03% w/v NaCl in demineralized water (29.03 g/100 ml) and 1% Tween 20 (Merck Sigma Aldrich, Darmstadt, Germany) was used as the starting concentration (Quan et al. 2017). A five-fold dilution in demineralized water was also prepared.

### Coating procedures

The salt solutions were applied to the test material by using two coating procedures that could be accessible in a home environment: spraying and dipping. For spray coating, a system consisting of a mini-spray valve (Nordson EFD781) mounted on an automated robot (Janome JR2304) was used to control the amount of salt deposition with the following defined parameters: speed of deposition, 40 mm/s; distance between spray head and test materials, 40 mm; pressure on the cartridge containing the salt solution, 0.4 bar; and deposition pattern, straight parallel lines 4 mm apart. The valve aperture controlling the flow (stroke) was set at 3 or 5, resulting in salt concentrations of 2.13 and 11.03 mg/cm^2^ on the test fabrics labeled Spr S3 and Spr S5, respectively.

For dip coating, a five-fold diluted salt solution (5.81% NaCl; 0.2% Tween 20) was used with an automated dip coater (KSV Nima medium, Biolin Scientific). The automatic system allowed the controlled full immersion of the fabrics for 3 s followed by withdrawal at a constant speed of 100 mm/min. The fabric samples were then suspended vertically for 30 min to drain and dry before storage. The dip-treated test material was labeled Dip Dil5 × with an estimated salt concentration of 10 mg/cm^2^.

Salt distribution and crystal aspect on the coated fabrics were checked via scanning electron microscopy (FEI Scios2, ThermoFisher Scientific, Waltham, MA, USA) in a low vacuum mode and energy-dispersive X-ray spectroscopy (X-Max 50 mm^2^ detector; Oxford Instruments, High Wycombe, UK; AZtec software control).

### Epithelial tissues

Primary human nasal epithelial cultures from a pool of donors were provided by Epithelix Sàrl (MucilAir™ pool of donors, article reference EP02MP, Epithelix Sàrl, Geneva, Switzerland) and handled in accordance with the manufacturer’s instructions. MucilAir™ is a 3D in vitro cell model of the human airway epithelium cultured at the air–liquid interface. These epithelia are fully mature and functional.

### Generation of virus stock solution

We used SARS-CoV-2 that was synthetically constructed using a yeast cloning system (Thao et al. 2020). The full-length sequence of SARS-CoV-2 (wild-type subtype) was confirmed by sequencing. SARS-CoV-2 was propagated in Vero E6 cells for 48 h, and, afterward, supernatant was centrifuged at 500×*g* for 5 min, aliquoted, and stored at − 80 °C until it was used for the infection assays. The viral titer of the stock was determined via a 50% tissue culture infective dose (TCID_50_) assay on Vero E6 cells. A 96-well plate was seeded with 2.0 × 10^6^ cells, i.e., 20,000 cells per well, 24 h before viral infection. The virus stock was serially diluted at 1:10 and incubated for 72 h. After incubation, the medium was removed, and the cells were fixed and stained with crystal violet. The titer was determined in accordance with the methods of Spearman–Kärber (Hierholzer and Killington 1996). The virus titer in the produced viral stock resulted in a titer of 4.0 × 10^5^ TCID_50_/ml. VeroE6 cells were cultured in Dulbecco’s modified minimal essential medium (DMEM) supplemented with 10% heat-inactivated fetal bovine serum, 1% nonessential amino acids, 100 µg/ml streptomycin, 100 IU/ml penicillin, and 15 mM HEPES (Gibco; Thermo Fisher Scientific).

### Infection assay

Three salt-coating conditions were assessed: two sprays, Spr S3 and Spr S5, and one dip, Dip Dil5×. Triplicates of each condition were prepared. Triplicates of untreated fabric samples, handled in the same way as the salt-coated samples, were used as the noncoated control. SARS-CoV-2 virus stock (50 µl) with a titer of 4.0 × 10^5^ TCID_50_/ml, was placed drop by drop on each of the test fabrics at room temperature for 10 min. After exposure, the virus particles were retrieved from the mask material by adding 500 µl of MucilAir™ medium, mixing via a few pipetting movements, and incubating at room temperature for 5 min. The obtained resolved virus solutions were immediately used for infection assays on primary human nasal epithelial cultures, and the leftover of the virus suspension was stored at − 80 °C for the subsequent titration and genome copy quantification.

Prior to infection, each insert was washed apically with the culture medium for 10 min. Three inserts were used per treatment group, *i.e*., three technical replicates. For inoculation, 200 μl of the virus-containing solution was applied to the apical side of the cultures and incubated at 33 °C in a humidified incubator (chamber humidity of 95% RH ± 5% RH) with 5% CO_2_ for 3 h. Subsequently, the inserts were washed rapidly and apically with the culture medium thrice to remove unbound virus particles.

For post-infection viral quantification, an apical wash with 200 μl of MucilAir™ culture media at 33 °C for 20 min was collected after 3, 24, 72, and 144 h of incubation. The collected apical liquids were stored at − 80 °C. Titration and virus genome copy number quantification were performed.

The following controls were included: virus control (20 µl of a virus stock of 4.0 × 10^5^ TCID_50_/ml in 180 µl MucilAir™ medium placed onto the apical side of the cultures), noninfected control (Mock exposed to 200 µl of culture medium on the apical side), antiviral control with remdesivir (180 µl of 10 μM remdesivir dissolved in DMSO, and 20 µl of 4 × 10^5^ TCID_50_/ml virus stock), control with DMSO alone, and control with Triton-X-100 (0.1% in MucilAir™ medium).

### Titration of progeny virus

The titer of the apical washes (3, 24, 72, and 144 h post-infection) and the titer of the retrieved viral solutions from the mask material were determined using a plaque assay in a 24-well format in PFU per milliliter. Vero E6 cells were seeded in a 24-well plate at a density of 2.0 × 10^5^ cells/ml 24 h before titration. After the cells were washed once with phosphate-buffered saline and the medium was changed, the cells were inoculated with apical washes containing viruses and serially diluted in a cell culture medium at 1:10 dilution. After 1 h of incubation, the inoculum was removed and subsequently overlaid with 1:1 mixture of 2.4% methylcellulose and 2 × DMEM supplemented with 20% fetal bovine serum, 200 IU/ml penicillin, and 200 µg/ml streptomycin. After 48 h of incubation at 37 °C, the cells were fixed in 4% (v/v) neutral-buffered formalin (Formafix AG, Hittnau, Switzerland) and stained with crystal violet. The number of wells displaying cytopathic effects was scored.

### Biosafety and biosecurity

All experiments with SARS-CoV-2 were performed in a BSL3 containment. A permit was issued by the responsible Swiss governmental authority for inactivation activities with SARS-CoV-2.

### Genome copy quantification

Apical washes (20 µl) were used for viral RNA extraction with an E.Z.N.A. viral RNA kit (Omega Biotek, Norcross, USA). Viral RNA was quantified via quantitative RT-PCR (TaqMan Fast Advanced Master Mix, Thermo Fisher Scientific) by using 5 µl of viral RNA with Mastermix and the specific SARS-CoV-2 primers pWhSF-E-F21 (5′-ACAGGTACGTTAATAGTTAATAGCGTACTTCT-3′) and pWhSF-E-R22 (5′-ACAATATTGCAGCAGTACGCACA-3′) and one probe with FAM-MGB-Q530 reporter-quencher dyes. The E gene of SARS-CoV-2 genome was used to design the primers. Four dilutions of a known concentration of SARS-CoV-2 and the controls for RNA extraction and RT-PCR were included, and the plates were run on QuantStudio™ 7 Flex PCR Detection System (Applied Biosystems, Thermo Fisher Scientific). C_t_ data were reported relative to the standard curve, corrected with the dilution factor, and presented as genome copy number per milliliter on the graphs.

### Determination of epithelial integrity

Transepithelial electrical resistance (TEER) was measured to verify that all the selected inserts satisfy the internal quality control standards (TEER > 200 Ω·cm^2^) (Boda et al. 2018).

The integrity of the MucilAir™ epithelia was determined 24, 72, and 144 h post-infection by measuring the TEER of the tissues with an EVOM2™ voltohmmeter (World Precision Instruments UK, Stevenage, UK).

### Data analysis

TEER values and virus replication performances between the test materials were statistically compared by using the R *t* test function and two-sample Student’s *t* test (two-tailed) with Welch modification to the degrees of freedom; the null hypothesis was that the true means of two groups were identical (Ripley 2001). These statistical comparisons were predefined based on scientific knowledge from literature. Data with *p* < 0.05 were considered significant, indicating that the null hypothesis should be rejected.

## Results

### Analysis of salt deposits

**Fig. 1:**
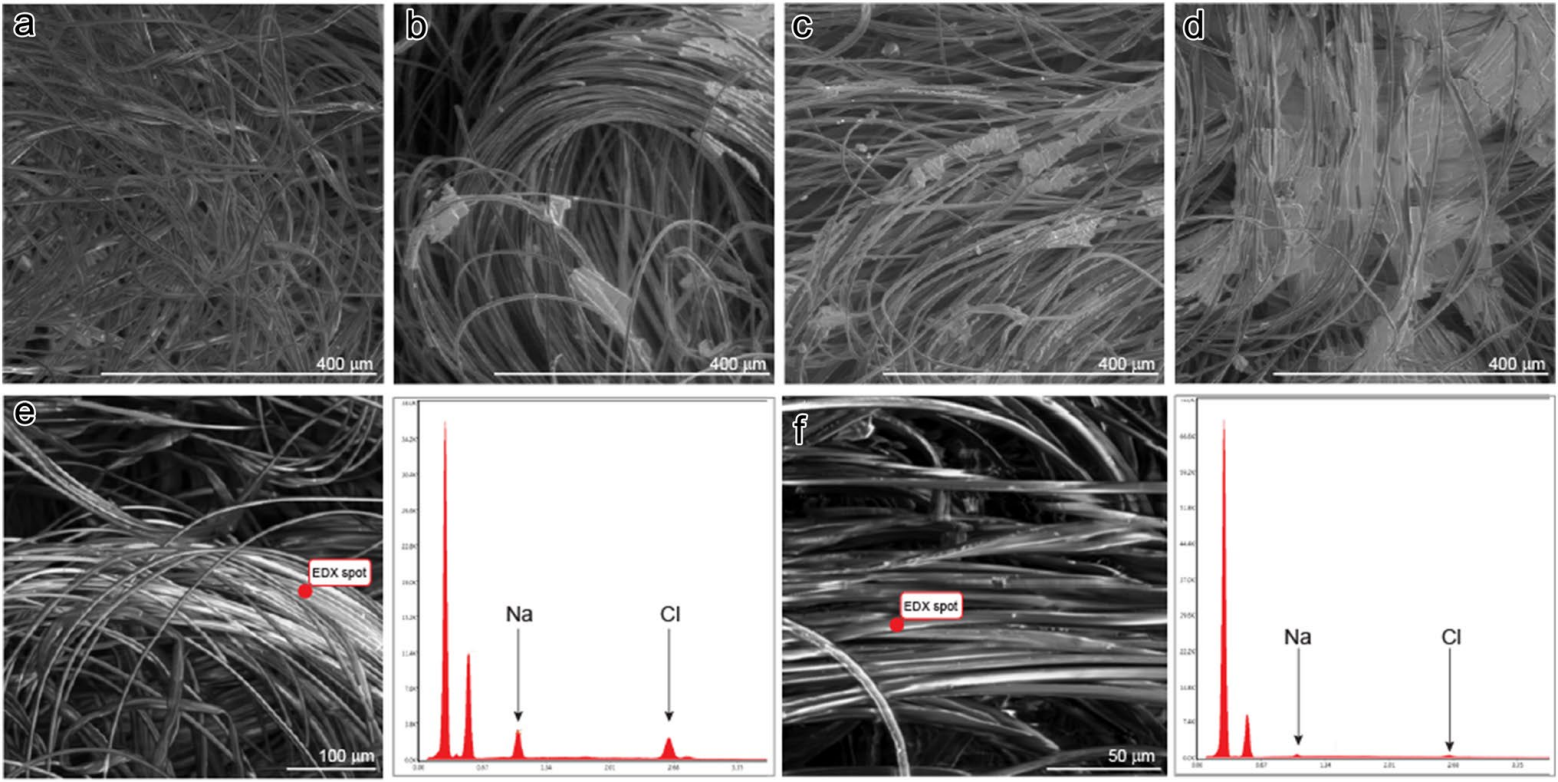
Scanning electron microscopy (SEM) and electron and energy-dispersive X-ray spectroscopy (EDX) analysis of fabric test materials. SEM images of **a** noncoated material, **b** Spr S3, **c** Spr S5, and **d** Dip Dil5×. EDX of **e** Spr S3 and **f** Spr S5

Salt deposition on the test material resulted in an uneven distribution of salt crystals of various sizes along the fibers of the fabrics (Fig. 1). Although the concentration of salt deposited on the test material increased according to spray strokes (2.13 mg/cm^2^ for Spr S3 and 11.03 mg/cm^2^ for Spr S5), scanning electron microscopy revealed similar patterns of salt crystal size and distribution in Spr S3 (Fig. 1b) and Spr S5 (Fig. 1c).

Energy-dispersive X-ray spectroscopy confirmed that salt was present on the fibers where larger salt agglomerates were not visible using scanning electron microscopy (Fig. 1e, f). Coating by dipping, even starting with a five-fold diluted salt solution, resulted in the formation of large salt agglomerates, often embedding several fibers in a crust-like appearance (Dil Dil5×; Fig. 1d) and affecting the mechanical properties of the material.

### TEER measurements in MucilAir™ epithelia after virus infection

Epithelial integrity can be monitored by TEER measurement. Undamaged MucilAir™ cultures typically yield TEER values of 200–600 Ω·cm^2^ (Boda et al. 2018). With severe losses of epithelial integrity, TEER values decrease below 100 Ω·cm^2^.

**Fig. 2:**
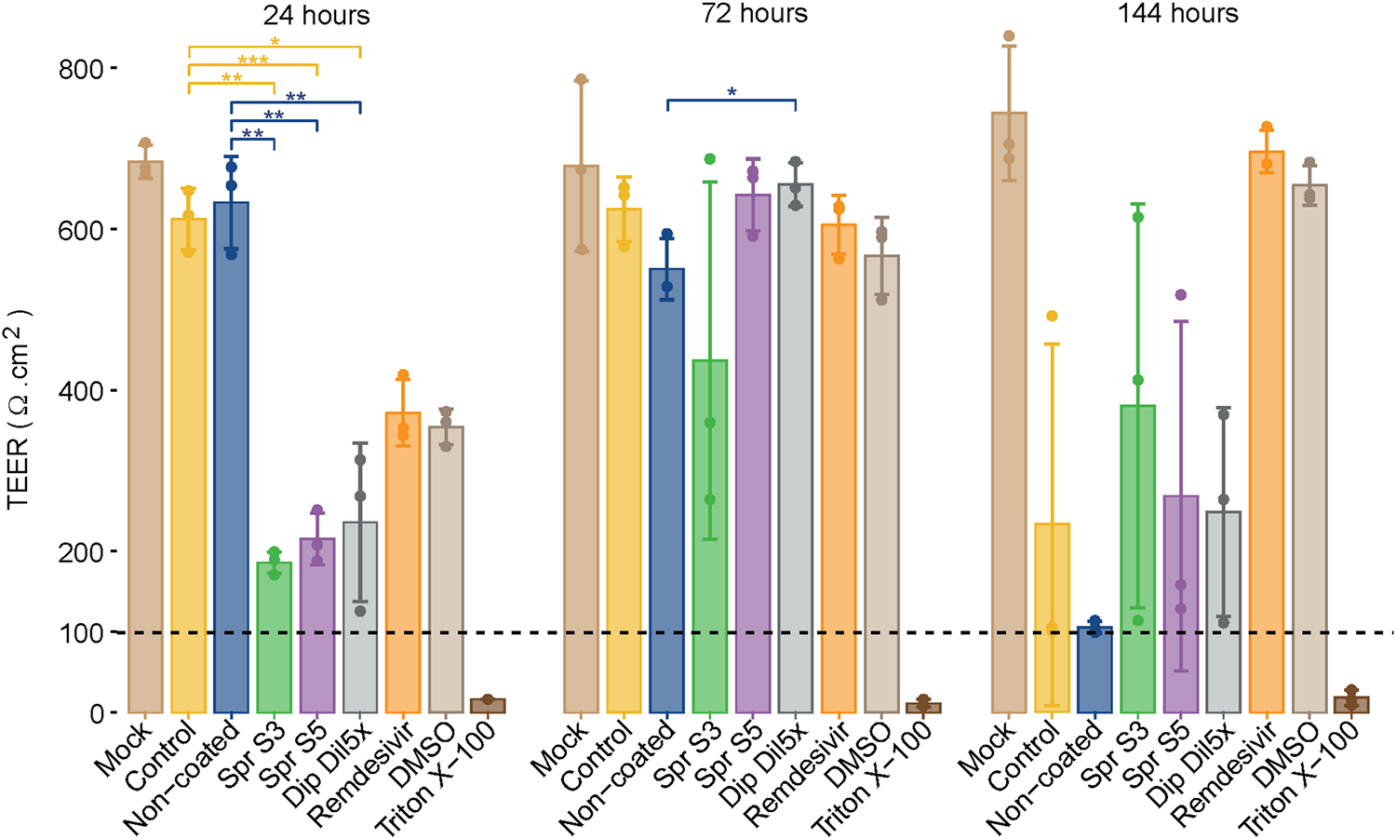
Transepithelial electrical resistance (TEER) measured in MucilAir™ epithelia after 24, 72, and 144 h of infection. Mean ± standard error; *n* = 3. The dotted line represents the 100 Ω·cm^2^ limit of tissue integrity. *p < 0.05, **p < 0.01, ***p < 0.001

TEER values in the noninfected mock remained between 600 and 800 Ω·cm^2^ during the experiment (Fig. 2). The inserts infected with the virus not exposed to the textile (control) or exposed to the noncoated textile maintained the TEER values in the same range as the mock until the 144th h of the measurement. At 144 h, the TEER values strongly decreased, indicating that viral replication might affect the epithelial structure over time.

In the control groups with remdesivir and DMSO, the TEER values were below 400 Ω·cm^2^ at the 24th h of measurement and likely influenced by DMSO in the samples as values returned to around 600 Ω·cm^2^ after 72 h. Interestingly, the TEER values in the cultures from the test conditions with the salt-coated material (Spr S3, Spr S5, and Dip Dil5×) were in the 200 Ω·cm^2^ range 24 h post-infection. These measured values were significantly lower than the TEER values of the control (Spr S3: *p* = 0.00382; Spr S5: *p* = 0.00020; Dip Dil5×: *p* = 0.01297), and the noncoated conditions (Spr S3: *p* = 0.00382; Spr S5: *p* = 0.00131; Dip Dil5×: *p* = 0.00742). At 72 h post-infection, the TEER measurements in the epithelia resulted in values comparable with those in the other treatment groups and mock. At 144 h post-infection, the TEER values declined under all coating conditions, and the trend was similar to that of the noncoated and virus controls. However, for all treatment groups and time points, the TEER values remained above the 100 Ω·cm^2^ threshold, showing that no severe loss of tissue integrity occurred during the experiment.

### Virus titration

**Fig. 3:**
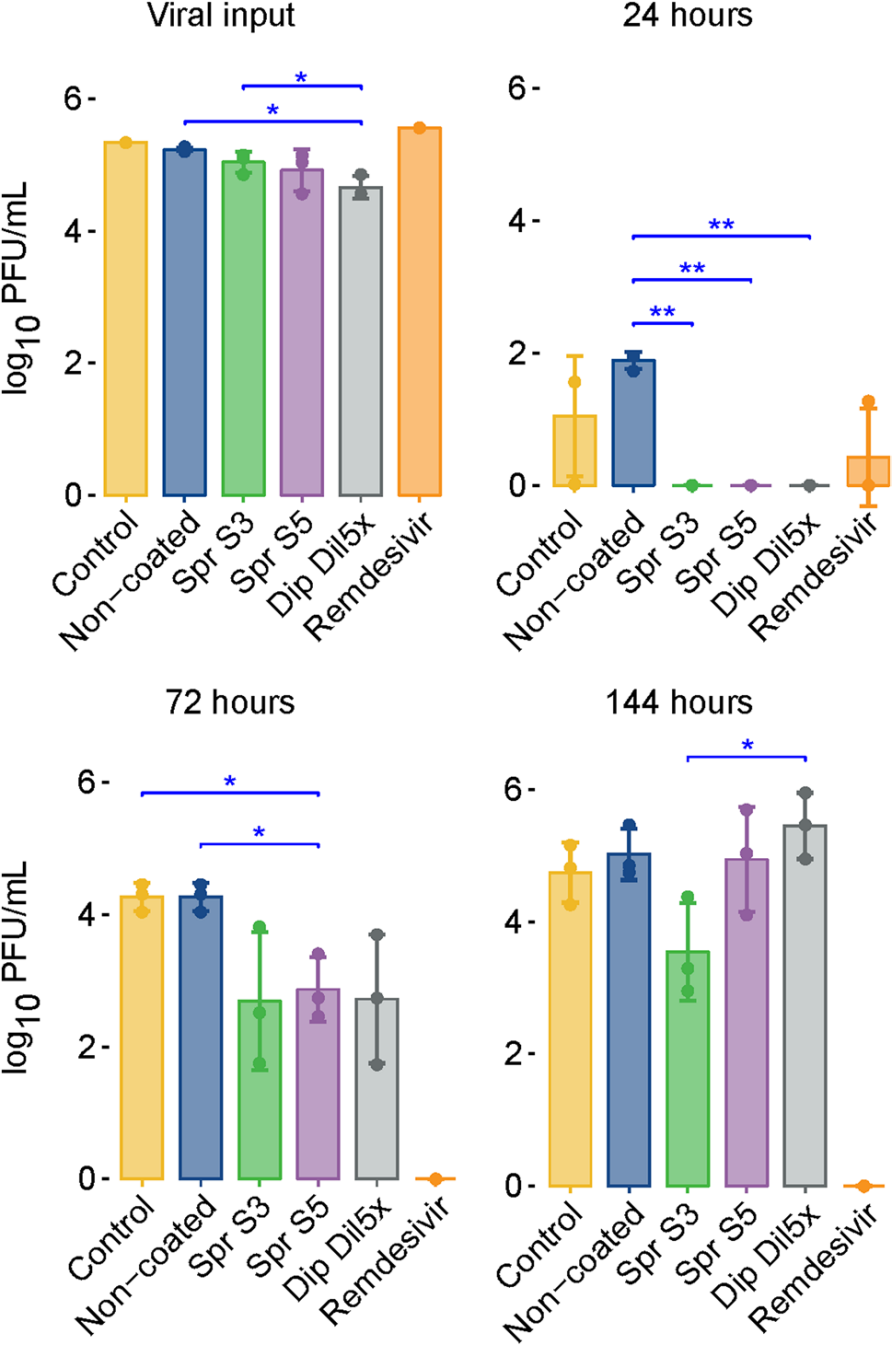
Viral titers from the viral inputs (virus collected after incubation with textiles or for cell infection with remdesivir or control medium) and from apical washes collected at 24, 72, and 144 h post-infection of the MucilAir™ epithelia. The titers were displayed as log_10_ PFU per milliliter. The titers are shown as mean values of three inserts, and error bars represent standard errors. *p < 0.05 and **p < 0.01

Progeny virus was quantified by titrating it in the retrieved viral solution and apical washes at 3, 24, 72, and 144 h post-infection (Fig. 3). The viral loads were determined via the plaque assay. Contact with the salt-coated material decreased the progeny virus load in the retrieved viral solution (viral input) compared with that in the noncoated textile. Viral titers were significantly lower in Dip Dil5× than in the noncoated group (*p* = 0.0275).

Virus titers remained below or close to the limit of detection of the assay in all test groups 3 h post-infection (results not shown). After 24 h of infection, virus titers were significantly lower in the three salt-coated samples than in the noncoated group (*p* = 0.00149). After 72 h of infection, live virus could be detected in all samples, but the viral titers in all samples from the salt-coated material were clearly reduced and significantly lower in Spr S5 than in the control and noncoated groups (*p* = 0.0235).

In summary, all coated materials showed antiviral effects with average log_10_ reductions of 1.58, 1.40, and 1.55 for Spr S3, Spr S5, and Dip Dil5×, respectively, compared with those of the noncoated sample. After 144 h of infection, viral titers measured in Spr S5 and Dip Dil5× were the same as those in the control and noncoated materials. The antiviral effect seemed to persist in Spr S3. Live virus could not be detected in the remdesivir group at 72 and 144 h post-infection.

### Genome copy quantification

**Fig. 4:**
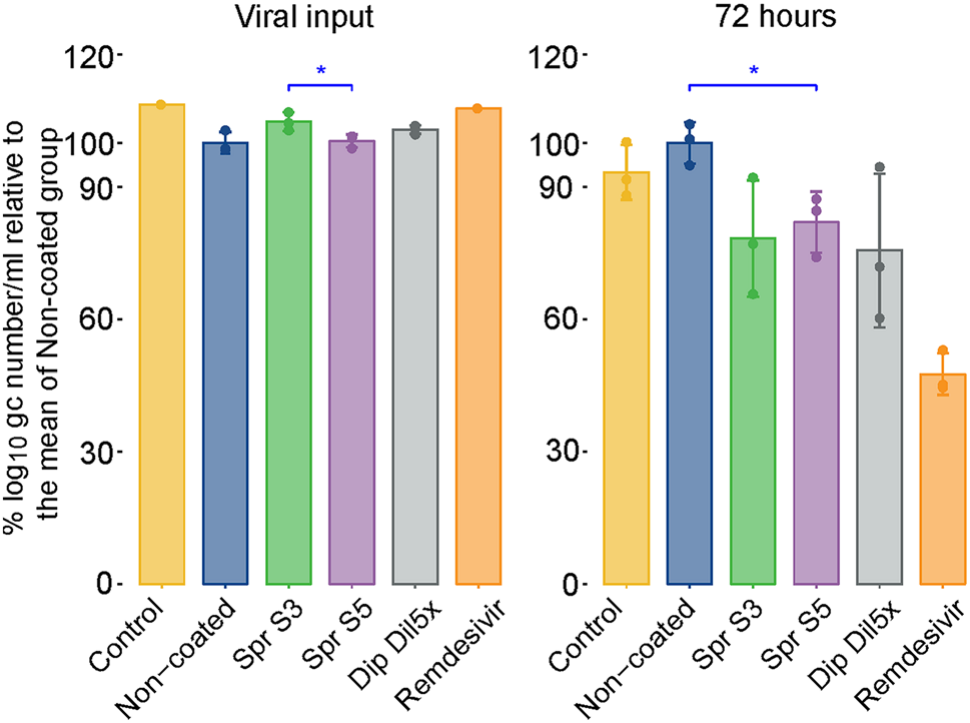
SARS-CoV-2 genome copy (gc) quantification in the medium after contact with coated or noncoated textiles (viral input and from MucilAir™ epithelial apical washes collected at 72 h post-viral infection. Data are expressed as percentage relative to the mean of log_10_ gc number/milliliter quantified in samples under the infected noncoated treatment condition, which was normalized to 100%. Genome copy values are shown as mean values of three replicates, and error bars represent standard errors. The viral inputs of the control medium (control) and remdesivir treatment groups were 20 µl of a SARS-CoV-2 virus stock with a titer of 4 × 10^5^ TCID_50_/ml. The medium and remdesivir viral inputs were used to infect three cell inserts. *p < 0.05

In addition to live virus titration, genome copy number quantification via qPCR was performed in the following: virus solution retrieved after contact with the coated or noncoated textiles, medium, remdesivir controls (viral input), and apical washes collected from the epithelial cell cultures at 72 h post-infection (Fig. 4). Genome copies were quantified to assess whether they were correlated with the detected live virus.

The genome quantification in the viral inputs did not result in the same reduction after contact with the salt-coated textile relative to the noncoated material, as previously seen in the viral titrations, indicating that the genomic material from the inactivated virus was still present in the viral inputs.

At 72 h post-infection, the virus was detected in apical washes from all treatment groups. As observed in titrations of the live virus, the three salt-coating treatments had a clear antiviral effect, resulting in a decrease in viral genome copy numbers compared with that of the noncoated group. Log_10_ reductions were in the same order of magnitude between genome copy quantification (1.48, 1.23, and 1.66 for Spr S3, Spr S5, and Dip Dil5×, respectively) and viral titers (see “Virus titration”). Genome copy numbers were significantly reduced in the samples collected from the Spr S5 coating treatment group compared with that of the noncoated group (*p* = 0.0259). In the apical washes, no discrepancy was observed between genome copy quantification and viral titers for the salt-coating treatments, implying no additional long-term effect on virus infectivity. Viral genome copy numbers in remdesivir were still clearly detectable, while viral titers were below the limit of detection in the PFU assay (Fig. 3).

## Discussion

This study evaluated the antiviral effect of salt deposited on reusable face mask materials against SARS-CoV-2 by using a well-standardized human 3D airway epithelium as an in vitro infection model (Pizzorno et al. 2020). The antiviral effect of salt coatings has been demonstrated against influenza A H1N1 virus (Quan et al. 2017) and pig *Alphacoronavirus* 1 (Tatzber et al. 2021) after deposition on a melt-blown material from disposable surgical masks. Here, we wanted to extend these findings to a material suitable for the manufacture of reusable cloth masks (Konda et al. 2020; Guha et al. 2021; Stan et al. 2021) against the agent of the current COVID-19 pandemic. Three salt-coating conditions (two with spray application and one with dip deposition) were tested and selected on the basis of the results obtained in the same epithelial cell system with influenza A H3N2 virus (Schorderet Weber et al. 2022).

The three salt-coating test conditions significantly slowed down viral replication in the airway epithelial cell cultures, as demonstrated by a clear reduction in the PFU in samples collected from cell apical washes after 24 h and 72 h post-infection. Viral titers generated from virus collected 24 h post-inoculation were below the assay detection limit in Spr S3, Spr S5, and Dip Dil5x samples, while viral titers in the virus control and the noncoated test group were clearly detectable. The effect was still marked at 72 h post-infection with log_10_ PFU reductions of about 1.5 in the salt-coated samples compared with that in the controls. According to (SeyedAlinaghi et al. 2022), the minimum infective dose for SARS-CoV-2 in human was estimated to be in the range of a few hundreds. Our results suggest that the salt-coated layer would drop virus loads below this threshold for at least 24 h after contact with virus-infected droplets and would help preventing contamination during mask wearing or handling. Virus titers in the salt-treated samples compared with the virus titers of the controls only 144 h post-infection. The quantification of genome copies in apical washes collected at 72 h post-infection confirmed the reduction in virus replication in these samples. As previously observed with Influenza H3N2 (Schorderet Weber et al. 2022), no clear negative correlation was observed between antiviral activity and salt quantity deposited on the test fabric, providing salt concentrations were above a certain threshold (> 0.6 mg/cm^2^). The coating conditions selected for the present study had all demonstrated antiviral activity against H3N2.

As previously reported (Quan et al. 2017; Rubino et al. 2021), the antiviral effect of salt is due to local dissolution followed by rapid recrystallization, i.e., a dynamic nucleation process, leading to mechanical disruption and acute osmotic stress of the viral envelope (Choi et al. 2015). As nucleation occurs over a wide range of concentrations, this effect is less dependent on the overall salt concentration in solutions. In the present study, although the volume of the virus-infected medium (50 µl) applied to 1 cm^2^ piece of fabric could dissolve most of the coating, the physicochemical processes that took place during the 10 min exposure were sufficient to damage the virus. During exposure, liquid diffusion onto the fibers of the material and evaporation contributed to the increase in the nucleation rate, and viral particles likely serve as nucleation centers. In general, the composition of salt in coatings may be less important than its ability to form crystals in aqueous solutions; a similar antiviral activity is observed in potassium chloride, potassium sulfate (Rubino et al. 2021), and sodium dihydrogen phosphate (Lee et al. 2021).

The TEER values measured at 24 h post-infection were significantly lower in the epithelial inserts that received the viral input exposed to the salt-coated material than in the control inserts (viral control and noncoated). The salt concentration in the viral inoculates ranged between 1.3 and 2.9% and might have influenced the viral intake by epithelial cells. A previous study reported a similar observation in MucilAir™ epithelia after contact with a 2.6% hypertonic salt solution (Huang et al. 2019). TEER values transiently decreased compared with those in the isotonic saline control and returned to normal after 3 days of the experiment. No tissue damage or increase in tissue permeability was observed, and changes in epithelial TEER were likely attributed to the activation of numerous sodium and chloride ion channels present in such type of epithelium (Hollenhorst et al. 2011). If salt did not damage the cell epithelia, could it have prevented or slowed down the entry and replication of SARS-CoV-2 into cells? The previous study using influenza virus A H3N2 as infection model revealed that incubation of the virus with NaCl solutions at concentrations of up to 35% did not affect virus replication in MucilAir™ cells although the TEER values comparably decreased in the presence of the highest salt concentration in the viral inoculate (2.6%) (Schorderet Weber et al. 2022). Similarly, in a system involving SARS-CoV-2, virus preincubation with salt concentrations up to 1.7% did not alter the success of virus replication in Vero cells (Machado et al. 2021).

Therefore, the low viral titers observed particularly after 24 and 72 h of infection in the cell cultures for Spr S3, Spr S5, and Dip Dil5x salt-coating conditions could be solely attributed to the contact of the virus particles with the coated material, triggering viral envelope disruption through salt dissolution and nucleation. Tween 20, also present in the salt coating, is a mild membrane solubilizer and permeabilizer (Johnson 2013) that affects the survival of enveloped viruses (Asculai et al. 1978). Although solutions with up to 0.5% Tween 20 have no influence on SARS-CoV-2 titers (Welch et al. 2020), higher concentrations, such as those encountered in the coating deposited on the fabric, may have facilitated the entry of salt into the virus envelope causing osmotic stress and envelope disruption during salt nucleation (Schorderet Weber et al. 2022).

In conclusion, the in vitro bioassay using human lung epithelia proved to be suitable for the assessment of antiviral coating effective against SARS-CoV-2 and could be used to test other types of antiviral face masks in the future. The antiviral effect of salt coatings previously reported for the influenza virus A H1N1 (Quan et al. 2017) and H3N2 (Schorderet Weber et al. 2022) is also confirmed for SARS-CoV-2. The survival of virus particles was significantly reduced after contact with salt-coated materials. Thus, these results provided easy and inexpensive solutions for the enhanced protection against self-contamination and the improved sustainability of personal protective materials.

## Data Availability

The datasets generated and analyzed in the present study are available from the corresponding author on reasonable request.
